# Direction of discordance between PET/CT, EBUS-TBNA, and multidisciplinary tumor board nodal assignment in non-small cell lung cancer: A retrospective observational study

**DOI:** 10.1097/MD.0000000000050981

**Published:** 2026-10-09

**Authors:** Orkun Eray Terzi, Ezgi Demirdöğen, Nilüfer Aylin Acet Öztürk, Özge Aydin Güçlü, Ahmet Ursavaş

**Affiliations:** a Department of Chest Diseases, Bursa Yuksek Ihtisas Training and Research Hospital, Bursa, Turkey; b Department of Chest Diseases, Bursa Uludag University Faculty of Medicine, Bursa Uludag University Hospital, Bursa, Turkey.

**Keywords:** decision concordance, EBUS-TBNA, mediastinal lymph node staging, multidisciplinary tumor board, non–small cell lung cancer, PET/CT

## Abstract

Mediastinal nodal classification in non–small cell lung cancer (NSCLC) at the N0/N1–N2/N3 treatment-defining boundary guides therapeutic strategy and is commonly finalized in multidisciplinary tumor board (MDT) meetings. We compared positron emission tomography/computed tomography (PET/CT) and endobronchial ultrasound–guided transbronchial needle aspiration (EBUS-TBNA) against MDT assignment and quantified discordance at this threshold. We retrospectively analyzed 114 consecutive NSCLC patients who underwent both PET/CT and EBUS-TBNA before MDT review. Nodal stage was dichotomized as N0/N1 vs N2/N3. Because the MDT decision incorporates both modalities, agreement with MDT is reported as observed agreement and Cohen κ together with the direction of discordance, rather than as sensitivity and specificity. In the subgroup with surgical mediastinal staging, both modalities and the MDT category were compared with histopathology. MDT assigned N2/N3 in 84 patients (73.7%). Relative to the MDT category, PET/CT over-classified 16 patients (14.0%) and under-classified 9 (7.9%). EBUS-TBNA under-classified 15 of the 43 patients it classified N0/N1 (34.9%), and under-classification persisted among the 25 patients in whom every sampled station was diagnostic (8/25, 32.0%); 12 of these 15 assignments rested on clinical–radiological course rather than histopathology. Agreement with the MDT category was 85.1% (κ = 0.662) for EBUS-TBNA and 78.1% (κ = 0.389) for PET/CT; because malignant cytology was definitional for the MDT category, 67 of 97 concordant EBUS-TBNA classifications (69.1%) were determined by that rule and do not measure EBUS-TBNA performance. Against surgical histopathology in 18 patients, PET/CT classified 11 of 13 pathologically N0 patients as N2/N3 (specificity 15.4%; 95% CI, 4.3–42.2) and EBUS-TBNA identified 2 of 5 with pathological N2 disease (sensitivity 40.0%; 95% CI, 11.8–76.9). At the treatment-defining nodal boundary, the two modalities diverged from the multidisciplinary decision in opposite directions: PET/CT over-classified nodal status, whereas EBUS-TBNA under-classified in approximately 1 in 3 patients it had assigned N0/N1, including those in whom sampling was complete, although most of these assignments rested on clinical course rather than tissue. These findings are consistent with guideline recommendations that PET-positive mediastinal nodes require tissue confirmation and that a negative endosonographic result be interpreted alongside pretest probability and station coverage.

## 1. Introduction

In non–small cell lung cancer (NSCLC) without distant metastases, nodal category is a principal factor in selecting the initial treatment pathway: patients categorized N0/N1 are generally considered for upfront curative-intent resection, whereas N2/N3 disease usually triggers multimodality treatment or further assessment of resectability.^[1]^ Positron emission tomography/computed tomography (PET/CT) is widely used for initial staging, but nodal classification is subject to misclassification in both directions: false-positive uptake arises from granulomatous and inflammatory nodal disease, and false-negative results from small-volume metastases.^[2,3]^ These limitations underpin the need for minimally invasive tissue confirmation when nodal status is expected to alter management.

Contemporary guidelines recommend endosonographic staging – most commonly endobronchial ultrasound–guided transbronchial needle aspiration (EBUS-TBNA), with endoscopic ultrasound (EUS) when available – as the first-line invasive modality for mediastinal staging in NSCLC.^[4–8]^ Reported EBUS performance varies with the spectrum of nodal disease sampled, patient selection, and study endpoints: sensitivity is higher in cohorts enriched for radiologically suspicious mediastinal nodes, whereas negative predictive value falls as the pretest probability of occult N2/N3 disease rises in clinically localized cases.^[9–11]^ This residual uncertainty is clinically consequential because occult mediastinal metastases may be present even in patients with a radiologically and metabolically normal mediastinum, in whom systematic endosonographic staging still upstages a proportion of cases.^[12–15]^

In routine practice, final nodal categorization and treatment allocation are usually determined in multidisciplinary tumor board (MDT) meetings that integrate imaging, endosonographic findings, clinical context, and, when available, surgical pathology. Although surgical sampling remains the reference standard, it is not uniformly feasible in real-world pathways; MDT consensus therefore frequently functions as a pragmatic decision standard, although one that is not independent of the modalities it integrates.

Mediastinal staging has nonetheless been reported largely as a diagnostic accuracy problem. Quantified less often is how frequently each modality places a patient on the opposite side of the N0/N1 vs N2/N3 boundary from the final MDT category (boundary crossing), and in which direction that discordance runs.

Against this background, the primary aim was to quantify how closely PET/CT and EBUS-TBNA agree with the MDT-assigned binary nodal category (N0/N1 vs N2/N3), with emphasis on agreement at the treatment-defining boundary and the direction of discordance. Secondary aims were to describe boundary crossing between the two modalities and to explore clinical and radiologic factors associated with discordance between EBUS-TBNA and the MDT-assigned category. In the subgroup that proceeded to surgical mediastinal staging, an exploratory aim was to compare both modalities and the MDT category with surgical histopathology.

## 2. Methods

### 2.1. Patient selection

This study is reported in accordance with the STROBE (Strengthening the Reporting of Observational Studies in Epidemiology) statement for observational research. Patients aged 18 years or older in whom a diagnosis of non-small cell lung cancer was established, or who underwent EBUS-TBNA for the staging of non-small cell lung cancer, were eligible. This retrospective, single-center observational study included consecutive patients who underwent endobronchial ultrasound-guided transbronchial needle aspiration (EBUS-TBNA) for lung cancer diagnosis and/or mediastinal staging between April 29, 2016, and February 3, 2023, at the Department of Pulmonary Diseases, Bursa Uludağ University Faculty of Medicine. All study variables were abstracted from routinely generated clinical, imaging, procedural, and pathology records; no study-specific investigations were performed, and no additional patient contact took place. Of 303 patients screened, 142 (46.9%) were excluded because follow-up or diagnostic confirmation data (n = 80) or radiological imaging (n = 62) were unavailable; a further 47 were excluded for a final diagnosis other than NSCLC (small cell lung cancer, 31; extra-thoracic malignancy, 11; sarcoidosis, 5). Because these exclusions favor patients managed to a documented treatment decision within the institution, the analytic cohort is likely enriched for patients who reached a recorded MDT assignment, which would tend to increase the apparent concordance between the modalities and the board. All variables used in the analyses were complete for the 114 patients included. No a priori sample size calculation was performed; all eligible patients treated during the study period were included, and the resulting sample size determined the precision of the estimates reported. The patient selection process is summarized in Figure 1. This retrospective study was approved by the Clinical Research Ethics Committee of Bursa Uludağ University Faculty of Medicine (decision no. 2021-8/30). The requirement for informed consent was waived because of the retrospective design and the use of de-identified data, and the study was conducted in accordance with the Declaration of Helsinki.

**Figure 1.**
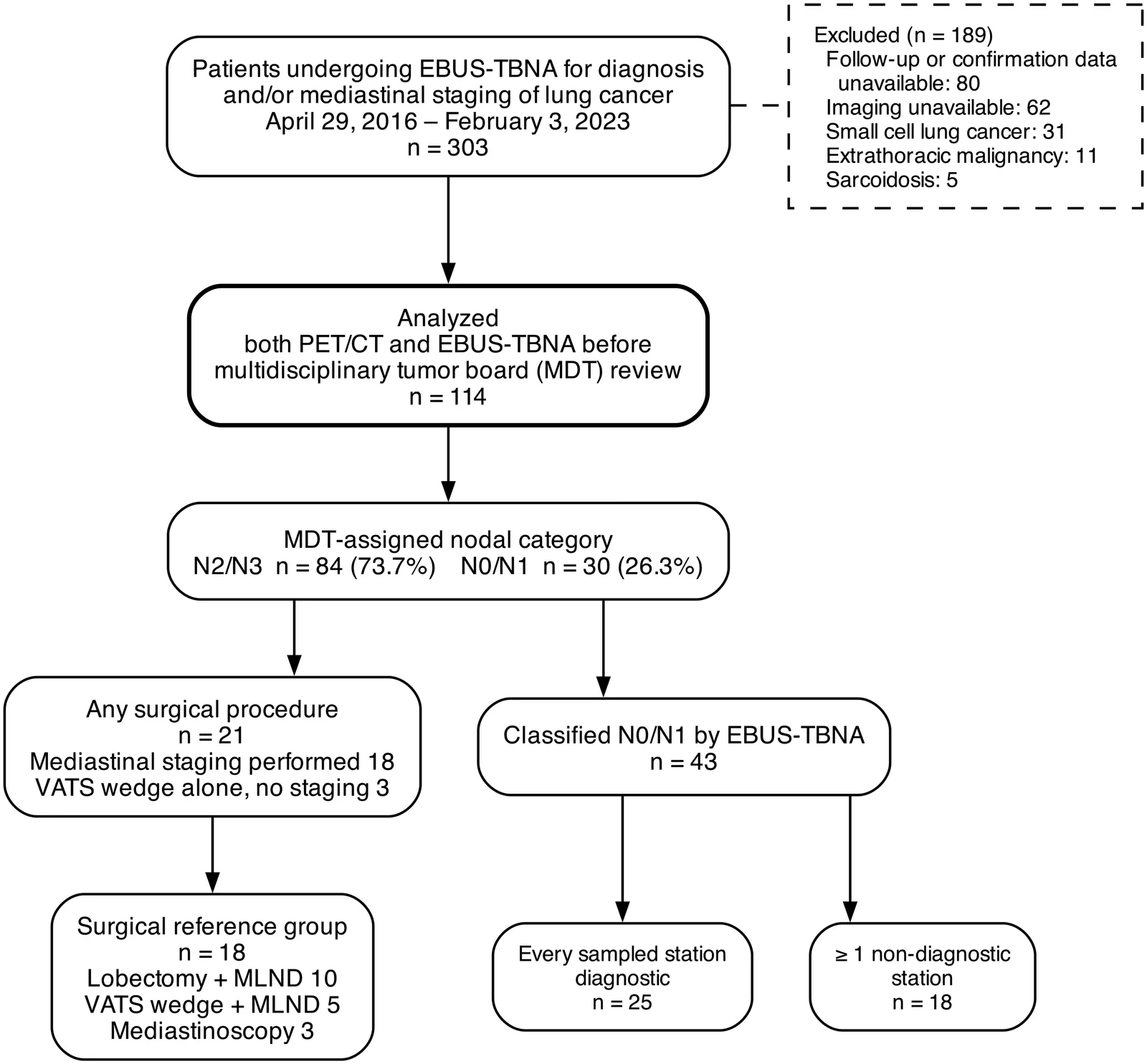
Flow of participants through the study. Of 303 patients who underwent endobronchial ultrasound–guided transbronchial needle aspiration (EBUS-TBNA) for the diagnosis and/or mediastinal staging of lung cancer between April 29, 2016 and February 3, 2023, 114 underwent both positron emission tomography/computed tomography (PET/CT) and EBUS-TBNA before multidisciplinary tumor board (MDT) review and were analyzed. The three patients who underwent video-assisted thoracoscopic surgery (VATS) wedge resection without mediastinal lymph node sampling were excluded from the surgical reference group but retained in the main analysis. MLND = mediastinal lymph node dissection.

### 2.2. Study procedures, MDT assessment, and data collection

All endobronchial ultrasound-guided transbronchial needle aspiration (EBUS-TBNA) procedures were performed under conscious sedation using a linear-array ultrasonic bronchoscope (BF-UC180F; Olympus, Tokyo, Japan) with a 7.5-MHz transducer. Prior to EBUS-TBNA, PET/CT was reviewed to localize the primary tumor and to define radiologic nodal status; lymph nodes with a maximum standardized uptake value (SUVmax) ≥ 2.5 were considered radiologically suspicious. During EBUS, the primary lesion (when applicable) and all accessible mediastinal, hilar, and interlobar lymph node stations were systematically assessed, and sampling was performed according to a standardized order (N3 → N2 → N1) to reduce cross-contamination and optimize staging accuracy. Aspirated material was prepared for direct smears and cell block, with additional liquid-based cytology when available, and was subsequently evaluated in the pathology laboratory. When surgical staging was performed, those findings were recorded.

Multidisciplinary tumor board (MDT) meetings were conducted in routine clinical practice and included pulmonology, thoracic surgery, medical oncology, radiation oncology, and radiology specialists. For decision-oriented analyses, the MDT-assigned nodal category was evaluated both as an ordinal stage (N0–N3) and dichotomized as N0/N1 vs N2/N3, reflecting the clinically relevant threshold guiding management pathways.

The following variables were recorded: age, sex, comorbidities, presenting symptoms, smoking status, histopathologic subtype, tumor location, primary tumor size and SUVmax on PET/CT, PET/CT-based T and N stage, metabolically active nodal stations on PET/CT, EBUS-assessed nodal size and EBUS-derived N stage, cytopathologic classification of EBUS samples (malignant, benign, or nondiagnostic), surgical staging results when available, and the final MDT-assigned nodal category.

To reduce measurement bias, cytopathological evaluation was performed blinded to imaging findings. Consecutive sampling was used to minimize selection bias.

### 2.3. Reference standard and adjudication of the MDT nodal category

The final nodal category was determined through a two-stage process at our institutional Pulmonary Oncology Multidisciplinary Tumor Board. At the first stage, cases were discussed together with the EBUS-TBNA result and assessed for the need for further invasive staging; patients for whom further staging was indicated were referred to thoracic surgery. At the second stage, the same board reviewed the case with the updated pathology and issued the stage and treatment decision.

Category assignment followed an explicit evidence hierarchy. In patients who underwent mediastinal lymph node dissection or sampling (n = 18; lobectomy with mediastinal lymph node dissection in 10, video-assisted thoracoscopic surgery (VATS) wedge resection with mediastinal lymph node dissection in 5, mediastinoscopy in 3), surgical histopathology took precedence over all other information; three further patients underwent VATS wedge resection without mediastinal staging and were excluded from the surgical reference group. These 3 patients remained in the main analysis and are included among the 96 patients without surgical mediastinal staging. In patients without surgical confirmation, a station yielding malignant cytology at EBUS-TBNA determined the nodal category. In patients with neither surgical confirmation nor malignant nodal cytology, the category was assigned by integrating PET/CT nodal SUVmax and short-axis diameter, station distribution relative to the primary tumor, the adequacy of sampling as documented in the procedure report, and the clinical–radiological course.

For lymph nodes initially considered malignant by the board, a reduction of nodal SUVmax to < 2.5 after chemotherapy or chemoradiotherapy was interpreted as treatment response, and the station was accepted as malignant; the threshold is the same as that used for baseline nodal positivity in this study.

A station sample was classified as nondiagnostic when it did not contain interpretable lymphoid material. Rapid on-site cytological evaluation was not used; institutional practice was to obtain at least three aspirations per station, and four when molecular testing was planned. Nondiagnostic samples did not establish nodal malignancy and therefore did not contribute to upstaging; the EBUS-TBNA nodal category was based on stations with an interpretable cytological result. Because a nondiagnostic station also could not exclude malignancy, such cases were revisited at the board in light of the PET/CT findings.

To quantify incorporation bias, PET/CT and EBUS-TBNA classifications were additionally analyzed against surgical histopathology in the subgroup of 18 patients with surgical mediastinal staging; this analysis was planned as exploratory.

### 2.4. Statistical analysis

All statistical analyses were performed using SPSS version 28.0 (IBM Corp., Armonk, NY, USA). Normality of continuous variables was assessed by visual inspection (histograms and Q–Q plots) and the Kolmogorov–Smirnov test. Continuous variables are presented as mean ± standard deviation (SD) when normally distributed and as median (minimum–maximum) when non-normally distributed. Categorical variables are summarized as counts and percentages, n (%).

Between-group comparisons were conducted using the independent-samples *t* test or the Mann–Whitney *U* test for continuous variables, and the chi-square test or Fisher’s exact test for categorical variables, as appropriate. Agreement between staging methods was assessed using observed agreement (%) and Cohen’s kappa (κ); for ordinal N categories (N0–N3), weighted κ (quadratic weights) was additionally considered. Agreement of each modality with the MDT category is reported as positive percent agreement (the proportion of patients assigned N2/N3 by the board who were also classified N2/N3 by the modality) and negative percent agreement (the corresponding proportion among patients assigned N0/N1). The complementary fractions in the modality-defined direction – the proportion of patients classified N2/N3, or N0/N1, by a modality who received the corresponding board category – are also reported; these are agreement fractions, not predictive values against an independent reference standard. Sensitivity, specificity, and predictive values are reported only for the subgroup with surgical mediastinal staging, in which histopathology provided an independent reference. Discordance is described by direction (over- vs under-classification relative to MDT). Patients whose EBUS-TBNA category differed from the MDT-assigned category were compared with concordant patients for demographic, clinical, and PET/CT variables using the χ^2^ or Fisher exact test and the Mann–Whitney *U* test, as appropriate; these comparisons were exploratory, were not adjusted for multiplicity, and are based on 17 discordant patients. Inter-modality agreement between PET/CT and EBUS-TBNA across four nodal categories (N0–N3) was also evaluated. Analyses were conducted on complete cases for the variables of interest. A two-sided *P* value < 0.05 was considered statistically significant. Confidence intervals for proportions were calculated using the Wilson method, and for Cohen κ from the asymptotic standard error. Because the MDT category incorporates PET/CT and EBUS-TBNA information, comparisons against it are decision-alignment metrics and are susceptible to incorporation bias.

## 3. Results

A total of 114 patients with non–small cell lung cancer (NSCLC) who underwent both PET/CT and EBUS-TBNA for mediastinal nodal evaluation were included in the final analysis. The median age was 64 years (range 21–82), and 96 patients (84.2%) were male. Histology was predominantly adenocarcinoma (63/114, 55.3%) and squamous cell carcinoma (46/114, 40.4%). On PET/CT, the primary tumor median long-axis diameter was 30 mm (range 12–90), short-axis diameter was 25.5 mm (range 10–73), and median SUVmax was 9.75 (range 1.8–38.88). The most frequently PET-positive nodal stations (SUVmax ≥ 2.5) were 4R (54.4%) and station 7 (43.9%) (Table 1). All primary variables were complete for the final analytic cohort (n = 114).

**Table 1:** Baseline characteristics, positron emission tomography/computed tomography findings, and endobronchial ultrasound–guided transbronchial needle aspiration nodal stage (n = 114).

Characteristic	n = 114
Age	64 [21–82]
Male sex	96 (84.2%)

Smoking history	
Current smoker	63 (55.3%)
Former smoker	34 (29.8%)
Never smoker	17 (14.9%)

Histology	
Adenocarcinoma	63 (55.3%)
Squamous cell carcinoma	46 (40.4%)
NSCLC, not otherwise specified	4 (3.5%)
Adenosquamous carcinoma	1 (0.9%)

PET/CT primary tumor (median [min–max])	
Long-axis diameter (mm)	30 [12–90]
Short-axis diameter (mm)	25.5 [10–73]
SUVmax	9.75 [1.8–38.88]

PET-positive lymph node stations (SUVmax ≥ 2.5) (Top 5) *	
Right lower paratracheal (4R)	62 (54.4%)
Subcarinal (7)	50 (43.9%)
Left interlobar (11L)	34 (29.8%)
Right hilar (10R)	32 (28.1%)
Right interlobar (11R)	30 (26.3%)
Other stations†	See footnote

Radiologic T stage (n, %)	
T1b	25 (21.9%)
T1c	17 (14.9%)
T2a	30 (26.3%)
T2b	8 (7.0%)
T3	12 (10.5%)
T4	22 (19.3%)

Radiologic N stage (n, %)	
N0	14 (12.3%)
N1	9 (7.9%)
N2	51 (44.7%)
N3	40 (35.1%)

EBUS-TBNA nodal stage (n, %)	
N0	38 (33.3%)
N1	5 (4.4%)
N2	46 (40.4%)
N3	25 (21.9%)

EBUS-TBNA = endobronchial ultrasound-guided transbronchial needle aspiration, NSCLC = non-small cell lung cancer, PET/CT = positron emission tomography/computed tomography, SUVmax = maximum standardized uptake value.

* Patients could have more than one PET-positive station; percentages are calculated over all 114 patients and therefore sum to more than 100%.

† Other stations (each <20%): 2R 18 (15.8%), 5 16 (14.0%), 4L 14 (12.3%), 3A 12 (10.5%), 8 11 (9.6%), 10L 7 (6.1%), 6 5 (4.4%), 2L 4 (3.5%), 9 2 (1.8%).

EBUS-TBNA nodal staging yielded N0 in 38 patients (33.3%), N1 in 5 (4.4%), N2 in 46 (40.4%), and N3 in 25 (21.9%). When collapsed into a binary nodal category, EBUS-TBNA classified 71/114 (62.3%) as N2/N3 and 43/114 (37.7%) as N0/N1. Radiologic nodal stage on PET/CT was N0 in 14 (12.3%), N1 in 9 (7.9%), N2 in 51 (44.7%), and N3 in 40 (35.1%); in binary terms, PET/CT classified 91/114 (79.8%) as N2/N3 and 23/114 (20.2%) as N0/N1. A median of 4 lymph node stations (range 1–6) was sampled per patient. Of 425 sampled nodal stations, 47 (11.1%) were reported as nondiagnostic; 32 patients (28.1%) had at least one nondiagnostic station, including 18 of the 43 patients (41.9%) classified as N0/N1 by EBUS-TBNA (Table 1).

Exact agreement between PET/CT and EBUS-TBNA across the four nodal categories (N0–N3) was observed in 57/114 patients (50.0%), corresponding to fair agreement (Cohen’s kappa = 0.284, quadratic weighted kappa = 0.300, *P* < .001) (Table 2). When dichotomized at the treatment-defining N0/N1–N2/N3 boundary (N0/N1 vs. N2/N3), PET/CT and EBUS-TBNA disagreed in 38/114 patients (33.3%), predominantly PET-positive/EBUS-negative crossing (29/114, 25.4%), with PET-negative/EBUS-positive crossing in 9 of 114 (7.9%).

**Table 2:** Positron emission tomography/computed tomography versus endobronchial ultrasound–guided transbronchial needle aspiration nodal stage (N0–N3): cross-tabulation (n = 114).

EBUS-TBNA N stage	PET/CT N0	PET/CT N1	PET/CT N2	PET/CT N3	Total
**N0**	9	3	17	9	38
**N1**	1	1	1	2	5
**N2**	3	5	28	10	46
**N3**	1	0	5	19	25
**Total**	14	9	51	40	114

EBUS-TBNA = endobronchial ultrasound-guided transbronchial needle aspiration, PET/CT = positron emission tomography/computed tomography.

Using the MDT-assigned nodal category as the decision framework, EBUS-TBNA showed positive percent agreement of 82.1% (69/84; 95% CI, 72.6–88.9) and negative percent agreement of 93.3% (28/30; 95% CI, 78.7–98.2), while PET/CT showed 89.3% (75/84; 95% CI, 80.9–94.3) and 46.7% (14/30; 95% CI, 30.2–63.9), respectively. In the modality-defined direction, the board assigned N0/N1 to 28 of the 43 patients classified N0/N1 by EBUS-TBNA (65.1%; 95% CI, 50.2–77.6) and N2/N3 to the remaining 15 (34.9%); among the 23 patients classified N0/N1 by PET/CT, 14 (60.9%; 95% CI, 40.8–77.8) were assigned N0/N1. The corresponding fractions among modality-positive patients were 69 of 71 (97.2%; 95% CI, 90.3–99.2) for EBUS-TBNA and 75 of 91 (82.4%; 95% CI, 73.3–88.9) for PET/CT, the former largely determined by the board’s use of malignant cytology to assign the category. Relative to the MDT category, PET/CT over-classified more patients than EBUS-TBNA (16 vs. 2), whereas under-classification was more frequent with EBUS-TBNA (15 vs. 9) (Tables 3 and 4).

**Table 3:** Agreement of endobronchial ultrasound–guided transbronchial needle aspiration and positron emission tomography/computed tomography with multidisciplinary tumor board–assigned nodal category (N2/N3 vs N0/N1) (n = 114). Decision-alignment analysis; MDT assignment is a pragmatic clinical comparator, not an independent reference standard. Panel A: EBUS-TBNA vs MDT; Panel B: PET/CT vs MDT.

	MDT N2/N3	MDT N0/N1	Total
Panel A			
EBUS N2/N3	69	2	71
EBUS N0/N1	15	28	43
Total	84	30	114

Panel B			
PET/CT N2/N3	75	16	91
PET/CT N0/N1	9	14	23
Total	84	30	114

Positive and negative percent agreement are reported instead of sensitivity and specificity because the MDT decision incorporated both PET/CT and EBUS-TBNA findings.

EBUS-TBNA = endobronchial ultrasound–guided transbronchial needle aspiration, MDT = multidisciplinary tumor board, PET/CT = positron emission tomography/computed tomography.

**Table 4:** Decision impact metrics relative to multidisciplinary tumor board–assigned nodal category (N2/N3 vs N0/N1).

Metric	EBUS-TBNA	PET/CT
**Concordant with MDT**	97 (85.1%)	89 (78.1%)
**Under-classified (N0/N1 → MDT N2/N3**)*	15 (13.2%)	9 (7.9%)
**Over-classified (N2/N3 → MDT N0/N1**)†	2 (1.8%)	16 (14.0%)
**Any discordance (under- + over-classified**)	17 (14.9%)	25 (21.9%)
**Net direction (under- minus over-classified**)‡	13 (11.4%)	−7 (−6.1%)
**Among modality N2/N3: MDT N2/N3 proportion**	69/71 (97.2%)	75/91 (82.4%)
**Among modality N0/N1: MDT N0/N1 proportion**	28/43 (65.1%)	14/23 (60.9%)

EBUS-TBNA = endobronchial ultrasound–guided transbronchial needle aspiration, MDT = multidisciplinary tumor board, PET/CT = positron emission tomography/computed tomography.

* Under-classified = modality N0/N1 while MDT assigned N2/N3.

† Over-classified = modality N2/N3 while MDT assigned N0/N1. Because malignant cytology was definitional for the MDT category, over-classification relative to MDT is constrained by design for EBUS-TBNA but not for PET/CT.

‡ Net direction is reported as (under-classified − over-classified).

EBUS-TBNA showed 97/114 (85.1%) overall concordance with MDT (κ = 0.662), whereas PET/CT showed 89/114 (78.1%) concordance (κ = 0.389) (Table 3).

Decision-impact metrics relative to MDT showed that for EBUS-TBNA, 15/114 (13.2%) were understaged and 2/114 (1.8%) were overstaged (any discordance 17/114, 14.9%). For PET/CT, 9/114 (7.9%) were understaged and 16/114 (14.0%) were overstaged (any discordance 25/114, 21.9%). Overall, EBUS-TBNA showed a net tendency toward understaging, whereas PET/CT showed a net tendency toward overstaging (Table 4).

Among the 25 EBUS-negative patients in whom every sampled station yielded an interpretable result, 8 (32.0%; 95% CI, 17.2–51.6) were assigned N2/N3 by the board, compared with 7 of 18 (38.9%; 95% CI, 20.3–61.4) among those with at least one nondiagnostic station. Of these 8 patients, 2 were confirmed by surgical histopathology and 6 by clinical–radiological course.

Surgical mediastinal staging was available in 18 patients (15.8%), of whom 5 (27.8%) had pathological N2 disease. In this subgroup, PET/CT identified all 5 patients with pathological N2 disease (sensitivity 100.0%, 5/5; 95% CI, 56.6–100.0) but classified 11 of 13 pathologically N0 patients as N2/N3 (specificity 15.4%, 2/13; 95% CI, 4.3–42.2). EBUS-TBNA identified 2 of the 5 (sensitivity 40.0%; 95% CI, 11.8–76.9) with a specificity of 84.6% (11/13; 95% CI, 57.8–95.7). The MDT-assigned category matched surgical histopathology in all 18 patients. The direction of discordance was similar to that observed against MDT assignment, although the subgroup was small, selected toward resectable disease, and had a lower N2 prevalence than the full cohort (27.8% vs 73.7%); these estimates are exploratory.

Among the 17 EBUS-TBNA cases discordant with MDT, 15 (88.2%) represented understaging and 2 (11.8%) represented overstaging. Evidence supporting the MDT-assigned nodal category comprised surgery/pathology confirmation in 5/17 (29.4%) and clinical–radiologic follow-up and/or treatment-response assessment in 12/17 (70.6%). Treatment-response support was defined as a posttreatment reduction of mediastinal lymph node SUVmax to < 2.5 after chemotherapy and/or chemoradiotherapy, consistent with MDT assignment (Table 5).

**Table 5:** Resolution of discordant endobronchial ultrasound–guided transbronchial needle aspiration cases relative to multidisciplinary tumor board–assigned nodal category (n = 17).

Discordant subgroup (relative to MDT)	n (%)	Evidence supporting MDT: Surgery/pathology n (% within subgroup)	Evidence: Follow-up/ treatment-response n (% within subgroup)
**EBUS understaging (EBUS N0/N1 → MDT N2/N3**)	15 (88.2%)	3 (20.0%)	12 (80.0%)
**EBUS overstaging (EBUS N2/N3 → MDT N0/N1**)	2 (11.8%)	2 (100.0%)	0 (0.0%)
**Total EBUS–MDT discordant**	17 (100%)	5 (29.4%)	12 (70.6%)

Evidence was categorized as surgery/pathology confirmation versus clinical-radiologic follow-up or treatment response. Treatment-response support reflects a posttreatment decrease of mediastinal lymph node SUVmax to < 2.5 after chemotherapy and/or chemoradiotherapy, consistent with MDT assignment. This criterion is based on PET/CT data and is therefore not independent of one of the index tests.

EBUS-TBNA = endobronchial ultrasound–guided transbronchial needle aspiration, MDT = multidisciplinary tumor board, SUVmax = maximum standardized uptake value.

In exploratory univariable comparisons, discordance between EBUS-TBNA and MDT nodal category was associated with PET/CT classification as N2/N3 (*P* = .022), while other baseline characteristics and PET/CT primary tumor metrics were broadly comparable between concordant and discordant groups; these comparisons were exploratory and hypothesis-generating (Table 6).

**Table 6:** Baseline and positron emission tomography/computed tomography characteristics according to concordance between endobronchial ultrasound–guided transbronchial needle aspiration nodal staging and multidisciplinary tumor board–assigned nodal category.

Characteristic	Concordant (n = 97)	Discordant (n = 17)	p value
Demographics			
**Male sex**	81 (83.5%)	15 (88.2%)	1.000
**Age, years**	64 [21–82]	64 [29–74]	0.509
**Any comorbidity**	62 (63.9%)	12 (70.6%)	0.784
**Respiratory symptoms at presentation**	86 (88.7%)	15 (88.2%)	1.000
**Smoking history (current or former**)	81 (83.5%)	16 (94.1%)	0.461
**Smoking exposure, pack-years**	40.00 [8.00–120.00]	40.00 [10.00–144.00]	0.799

PET/CT characteristics			
**Primary tumor location, upper lobe**	83 (85.6%)	14 (82.4%)	0.717
**Primary tumor short-axis diameter, mm**	25.00 [12.00–73.00]	32.00 [10.00–60.00]	0.251
**Primary tumor long-axis diameter, mm**	30.00 [12.00–90.00]	40.00 [18.00–70.00]	0.192
**Primary tumor SUVmax**	9.25 [1.81–24.10]	11.00 [4.20–38.88]	0.090
**Radiologic T stage, T3/T4**	28 (28.9%)	6 (35.3%)	0.578
**Radiologic N stage, N2/N3**	74 (76.3%)	17 (100.0%)	0.022
**Any nodal metastasis on PET/CT (N1/N2/N3**)	83 (85.6%)	17 (100.0%)	0.124

EBUS-TBNA = endobronchial ultrasound-guided transbronchial needle aspiration; MDT = multidisciplinary tumor board; PET/CT = positron emission tomography/computed tomography; SUVmax = maximum standardized uptake value.

## 4. Discussion

Mediastinal lymph node staging is central to NSCLC management because it directly determines treatment allocation and prognostic stratification. Although surgical staging provides histopathologic confirmation, its invasiveness and limited routine feasibility support contemporary stepwise pathways that combine noninvasive imaging with minimally invasive tissue sampling. Accordingly, PET/CT and endosonographic staging (EBUS with or without EUS) are routinely integrated into mediastinal assessment; however, these modalities may assign different nodal categories in the same patient, creating uncertainty precisely at the points where management changes.

In real-world practice, the MDT integrates PET/CT findings, endosonographic sampling results (EBUS with or without EUS), and clinical context to assign a pragmatic nodal category and determine the treatment pathway. Accordingly, beyond a purely diagnostic-accuracy framework, assessing inter-modality agreement, concordance with MDT-assigned nodal category, and decision impact – particularly the direction of discordance (under- vs. over-classification) – offers clinically actionable insight. In this study, we compared PET/CT- and EBUS-TBNA–based nodal staging in NSCLC to characterize agreement, MDT concordance, reclassification patterns at the treatment threshold, and how discordant cases were adjudicated within routine MDT decision-making.

In our study, mediastinal staging was intentionally interpreted through an MDT-centered lens, consistent with stepwise pathways in which PET/CT risk mapping is integrated with endosonographic tissue sampling when nodal status is expected to alter management. Within this framework, EBUS-TBNA showed higher concordance with the MDT-assigned nodal category (κ = 0.662; 85.1% agreement) than PET/CT (κ = 0.389; 78.1% agreement), while inter-modality agreement across N0–N3 remained only fair (κ = 0.284). When dichotomized at the treatment-defining N0/N1–N2/N3 boundary, PET/CT and EBUS-TBNA crossed the decision threshold in one-third of patients, predominantly due to PET-positive/EBUS-negative classification. Discordance was directional: PET/CT more often contributed to MDT-discordant over-classification (16 over-classified vs. 9 under-classified), whereas EBUS-TBNA discordance predominantly reflected under-classification (15 under-classified vs. 2 over-classified). These patterns align with prior prospective data showing stronger agreement for endosonographic approaches than for PET/CT when tissue confirmation is incorporated.^[16]^ Because MDT recommendations themselves show only moderate reproducibility across settings,^[17–19]^ our findings should be interpreted as quantifying modality alignment with real-world decision-making rather than estimating “true” diagnostic accuracy.

EBUS-TBNA has become a central modality for mediastinal staging because it enables minimally invasive tissue confirmation with high specificity when malignant nodal material is obtained. Early prospective series reported high sensitivity and near-perfect specificity, reflecting strong “rule-in” capability of positive samples.^[20]^ Subsequent evidence, however, has shown that sensitivity is context-dependent and may be substantially lower when EBUS is applied for systematic detection of radiologically occult N2/N3 disease in clinically cN0/cN1 patients; a systematic review and meta-analysis reported pooled sensitivity of 49% with specificity approaching 100% in this setting.^[11]^ Other systematic endosonographic staging series similarly report sensitivity/NPV estimates around 77%/84%, underscoring that negative results may retain residual uncertainty even in structured pathways.^[21]^ Because malignant cytology was definitional for the board’s category, EBUS-TBNA and the MDT assignment agreed by construction in most positive cases. The informative comparison is therefore confined to EBUS-negative patients and to the surgically staged subgroup. Only 28 of 43 patients classified as N0/N1 by EBUS-TBNA were assigned N0/N1 by the board (65.1%), in a decision context where N2/N3 prevalence reached 73.7%; among the 18 surgically staged patients, EBUS-TBNA missed 3 of the 5 with pathological N2 disease. Negative endosonography therefore warrants cautious interpretation when pretest probability remains high.

PET/CT is widely used as a noninvasive component of mediastinal assessment, providing whole-body staging and mapping suspicious nodal targets for confirmation. Guideline summaries emphasize that PET/CT improves nodal staging over CT alone but that tissue confirmation is required when mediastinal nodes are PET-positive or enlarged.^[2]^ In routine practice, specificity can be substantially lower due to false-positive FDG uptake in inflammatory or granulomatous conditions, as shown in prospective “real-life” data.^[16]^ PET/CT diverged in the opposite direction: it identified most patients whom the board assigned to N2/N3 (positive percent agreement 89.3%) but agreed poorly with the board’s N0/N1 assignments (negative percent agreement 46.7%; overall agreement 78.1%, κ = 0.389). These figures are not directly comparable with those for EBUS-TBNA, because only a positive EBUS-TBNA result was definitional for the board’s category. Against histopathology in the surgically staged subgroup, 11 of 13 pathologically N0 patients had been classified as N2/N3 on PET/CT. This profile supports a pragmatic stepwise workflow in which PET/CT triages suspicion and prioritizes targets, while endosonographic sampling stabilizes nodal assignment when management would change.

Discordance between EBUS-TBNA and the MDT-assigned nodal category occurred in 17 patients and predominantly reflected EBUS-TBNA under-classification relative to MDT (15/17, 88.2%). In the concordant–discordant comparison (Table 6), PET/CT classification as N2/N3 was the only factor significantly associated with discordance (100.0% vs 76.3%; *P* = .022), whereas primary tumor metrics and SUVmax showed only nonsignificant trends. A prospective study of patients staged N0/N1 by EBUS-TBNA and subsequently resected reported abnormal mediastinal appearance on CT/PET as the factor most strongly associated with false-negative endosonographic staging (odds ratio 7.77; 95% CI, 2.19–27.51), alongside the extent of satisfactory mediastinal sampling.^[22]^ Accordingly, when PET/CT remains suspicious for mediastinal metastasis, a negative EBUS result should prompt MDT review of station coverage and sampling adequacy, with consideration of selective additional confirmation or close follow-up. Given the limited number of discordant cases, these findings should be interpreted as exploratory and hypothesis-generating.

Discordant staging was adjudicated through routine-care pathways: among discordant EBUS-TBNA cases, MDT assignment was supported by surgery/pathology in 29.4% and by clinical–radiologic follow-up and/or treatment-response assessment in 70.6%. This mirrors real-world practice, where universal surgical verification is uncommon. Guidelines position endosonography as the first-line invasive test, but the role of additional confirmation after a negative result remains contested. The randomized MEDIASTrial reported that confirmatory mediastinoscopy can be omitted after negative systematic endosonography,^[23]^ whereas a systematic review and meta-analysis found that confirmatory video-assisted mediastinoscopy raised the negative predictive value from 79.2% to 91.8%.^[24]^ Taken together, these findings support selective rather than routine additional confirmation.^[1,5]^

A clinically important contributor to residual discordance is anatomical access. EBUS-TBNA does not sample subaortic (station 5) and para-aortic (station 6) nodes, and standard cervical mediastinoscopy has limited reach to the aortopulmonary (AP) window. When these stations are clinically relevant, alternative approaches (e.g., parasternal mediastinotomy or video-assisted thoracoscopic surgery–based sampling) may be required.^[2,8]^ In our study, station 5 malignancy identified in a surgically upstaged discordant case illustrates how AP-window disease can remain clinically consequential despite negative endosonography. Accordingly, suspected AP-window involvement – particularly in left-sided tumors – should prompt consideration of an approach that reaches stations 5 and 6 before a negative endosonographic result is relied upon.

A wider implication concerns studies that use a multidisciplinary decision as the comparator for staging tests. Where a single reference standard cannot be applied to every patient, composite or consensus standards are an established substitute, and the reference standard remains a recognized domain of bias in diagnostic accuracy research.^[25,26]^ Where the board’s rule makes a positive biopsy definitional, the biopsy modality cannot disagree with the board except when surgery overturns it, and its apparent agreement, κ, and positive percent agreement become properties of the rule rather than of the test. In our cohort this applied to 67 of the 97 concordant classifications (69.1%), and agreement outside those cells was 30 of 47 (63.8%), compared with 85.1% overall. Concordance with multidisciplinary assignment is therefore interpretable only where the adjudication rule is stated and the proportion of classifications determined by that rule is reported.

A key strength of the present study is the systematic integration of PET/CT, EBUS-TBNA, and – when indicated – surgical staging within an MDT framework, reflecting routine clinical practice and enabling assessment of how staging information translates into treatment decisions. All EBUS-TBNA procedures were performed by two experienced bronchoscopists, supporting procedural consistency and limiting operator-dependent variability. Cytopathological evaluation was performed blinded to imaging and clinical staging, reducing interpretive bias. Data abstraction followed predefined variable definitions and structured documentation, strengthening internal consistency; additionally, documenting adjudication pathways for discordant cases enhanced the decision-relevance of the dataset.

Because malignant cytology at EBUS-TBNA was definitional for the MDT-assigned category, the agreement we report between EBUS-TBNA and the board is largely a property of that decision rule, as set out above. A patient classified N2/N3 by EBUS-TBNA could be assigned N0/N1 only where surgical histopathology contradicted the cytology, which occurred in 2 patients, both in the surgically staged subgroup and none among the 96 patients without surgical staging. In both, station 4R was resected and found free of tumor; these are best interpreted as false-positive cytology results. Overall agreement of 85.1%, κ = 0.662 and positive percent agreement of 82.1% should therefore not be read as measures of EBUS-TBNA performance, and the contrast with PET/CT (κ = 0.389, negative percent agreement 46.7%) reflects the decision rule rather than relative specificity. Under-classification carried no such constraint – 12 of the 96 patients without surgical staging were classified N0/N1 by EBUS-TBNA and assigned N2/N3 by the board – and the surgically staged subgroup provides the only estimates in this study that are free of the constraint.

The route by which most under-classifications were established is not independent of one of the index tests. Of the 15 patients classified N0/N1 by EBUS-TBNA and assigned N2/N3 by the board, 3 were confirmed by surgical histopathology and 12 by clinical–radiological course, including the PET/CT-based treatment-response criterion. That criterion could support but never overturn an assignment of malignancy, since a failure of nodal SUVmax to fall did not reclassify a node. It was also categorical: no minimum relative decline was required, and posttreatment SUVmax values were reviewed at the board meeting and were not retained in the analysis dataset, so the margin by which nodes fell below the 2.5 threshold cannot be reported. A minimum follow-up period was not defined, and follow-up duration was not recorded. Under-classification by EBUS-TBNA relative to the board is therefore established through PET/CT-informed adjudication in four of five instances, and its magnitude may be overstated to the extent that PET/CT up-classification was itself unconfirmed.

In the 18 patients with surgical mediastinal staging, the board assignment reproduced histopathology in every case, which quantifies rather than resolves the incorporation bias: where a surgical result existed, it entered the board decision, and MDT-referenced estimates in that subset carry no independent information.

Our cohort was staged according to the eighth edition of the TNM classification. The ninth edition, effective from January 2025, separates ipsilateral mediastinal involvement into single-station (N2a) and multi-station (N2b) disease.^[27]^ Because this distinction divides precisely the subgroup in which resectability is debated,^[28]^ the N0/N1 vs N2/N3 dichotomy used here remains decision-relevant but does not capture station-count heterogeneity within N2.

Surgical confirmation was unavailable in 96 patients, so under-classification relative to the board rests on cytology and clinical course in most cases, with histological confirmation in a minority. Complementary esophageal staging (EUS or EBUS scope–guided transesophageal ultrasound [EUS-B]) was not routinely performed, and because stations 8 and 9 are not accessible by EBUS, this would be expected to inflate the under-classification we report.^[29]^ Intervals between PET/CT and EBUS-TBNA, and between EBUS-TBNA and surgery, were not retrievable and are not reported. Where these intervals were long, interval progression could account for part of the discordance we attribute to disagreement between modalities. Although patients were managed at a single center, PET/CT was performed at referring institutions with heterogeneous acquisition and reporting practices, which would widen rather than narrow the PET/CT–board discordance we report. The exclusion of 142 screened patients for unavailable data is likely to have enriched the cohort for patients who reached a recorded MDT assignment, which would tend to increase the apparent concordance reported here.

## 5. Conclusion

In this single-center cohort, PET/CT and EBUS-TBNA placed patients on opposite sides of the treatment-defining N0/N1–N2/N3 boundary in 38 of 114 patients (33.3%), and they diverged from the board’s assignment in opposite directions. PET/CT over-classified nodal status relative to the board in 16 patients and under-classified in 9, whereas EBUS-TBNA under-classified in 15 of the 43 patients it assigned N0/N1 (34.9%). Because malignant cytology was definitional for the board’s category, only the under-classification direction is free of the decision rule; against surgical histopathology in the 18 resected patients, the same directions were seen, with PET/CT classifying 11 of 13 pathologically N0 patients as N2/N3 and EBUS-TBNA identifying 2 of 5 patients with pathological N2 disease. These observations are consistent with guideline recommendations that PET-positive mediastinal nodes require tissue confirmation and that a negative endosonographic result be interpreted alongside pretest probability and station coverage. In routine practice, the size of that residual uncertainty is often unrecorded: 18 of the 43 patients (41.9%) classified N0/N1 by EBUS-TBNA had at least one nondiagnostic station, information available at the moment of the board decision. Recording sampling adequacy and station coverage in the board’s documentation would make it visible when the treatment pathway is chosen.

## Author contributions

**Conceptualization:** Orkun Eray Terzi, Ezgi Demirdöğen.

**Data curation:** Orkun Eray Terzi, Nilüfer Aylin Acet Öztürk, Özge Aydin Güçlü.

**Formal analysis:** Orkun Eray Terzi.

**Investigation:** Orkun Eray Terzi, Nilüfer Aylin Acet Öztürk, Özge Aydin Güçlü.

**Methodology:** Orkun Eray Terzi, Ezgi Demirdöğen.

**Project administration:** Orkun Eray Terzi, Ezgi Demirdöğen.

**Supervision:** Ezgi Demirdöğen, Ahmet Ursavaş.

**Validation:** Orkun Eray Terzi, Ezgi Demirdöğen, Ahmet Ursavaş.

**Visualization:** Orkun Eray Terzi.

**Writing – original draft:** Orkun Eray Terzi, Ezgi Demirdöğen.

**Writing – review & editing:** Orkun Eray Terzi, Ezgi Demirdöğen, Nilüfer Aylin Acet Öztürk, Özge Aydin Güçlü, Ahmet Ursavaş.
